# Aspirin or Ticagrelor in *Staphylococcus aureus* Infective Endocarditis: Where Do We Stand?

**DOI:** 10.3389/fcell.2021.716302

**Published:** 2021-10-07

**Authors:** Kirsten Leeten, Nicolas Jacques, Patrizio Lancellotti, Cécile Oury

**Affiliations:** ^1^Laboratory of Cardiology, Department of Cardiology, GIGA Institute, University of Liège Hospital, Liège, Belgium; ^2^Gruppo Villa Maria Care and Research, Maria Cecilia Hospital, Cotignola, and Anthea Hospital, Bari, Italy

**Keywords:** infective endocarditis, antiplatelet drugs, ticagrelor, aspirin, biofilm, *Staphylococcus aureus*

## Abstract

Infective endocarditis is a challenging disease with a high mortality and morbidity rate. Antibiotic prophylaxis is currently recommended in high-risk infective endocarditis patients. However, the use of antibiotics faces the challenge of a low efficacy and contributes further to the emerging infection rate by antibiotic-resistant strains, emphasizing the need for new therapeutic strategies. Platelets are essential in the initial phase of infective endocarditis, acting as first-line immune responders. During the first phase of disease, bacteria can interact with platelets and counteract platelet antimicrobial activities. Mechanistic *in vitro* and animal studies on the effect of aspirin on bacteria-platelet interactions and the prevention of vegetation development showed promising results. However, data from clinical studies on the outcome of infective endocarditis patients who were receiving medically indicated aspirin therapy remain controversial. Therefore, the benefit of antiplatelet agents in infective endocarditis prevention has been questioned. Besides aspirin, it has been discovered that the platelet P2Y12 receptor antagonist ticagrelor has antibacterial properties in addition to its potent antiplatelet activity. Furthermore, a recent study in mice and a case report remarkably indicated the ability of this drug to eradicate *Staphylococcus aureus* bacteremia. This review will focus on current knowledge on antibacterial activity of ticagrelor, compared to aspirin, pointing out main unanswered questions. The goal is to provide food for thought as to whether a prior ticagrelor therapy might be beneficial for the prevention of infective endocarditis.

## Introduction

Infective endocarditis (IE) is a life-threatening infectious disease, affecting the heart valves, or (bio-) prosthetic valve implants (Holland et al., 2016). The disease has been associated with a one-year mortality rate of around 30–40% (Liesenborghs et al., 2020). Gram-positive bacteria are the main instigators of IE, with *Staphylococcus aureus* (*S. aureus*) being the most prominent and virulent one (Werdan et al., 2014; Tong et al., 2015; Cahill and Prendergast, 2016; Holland et al., 2016; Liesenborghs et al., 2018; Habib et al., 2019). IE is characterized by the formation of a vegetation on the heart valve surface, consisting of bacteria, platelets, fibrin, and leukocytes (Moreillon and Que, 2004; Que and Moreillon, 2011; Liesenborghs et al., 2019). Disease initiation depends on the overall ability of bacteria to be cleared from the blood stream, to adhere to damaged or inflamed endothelium, and to bypass the host defense (Bayer et al., 1997).

Antibiotic prophylaxis is currently recommended in patients at high risk to develop IE (Wilson et al., 2007; Que and Moreillon, 2011). However, this further contributes to a new pandemic of antibiotic-resistant bacterial strains, emphasizing the need for additional strategies to prevent IE development (Que and Moreillon, 2011).

Data from *in vitro* and *in vivo* preclinical studies indicated reduced vegetation growth when the antiplatelet agent aspirin was used as prophylactic or adjunct therapy (Nicolau et al., 1995, 1999; Kupferwasser et al., 1999a, b, 2003; Veloso et al., 2015a, b). Several prospective and retrospective clinical studies have evaluated the ability of aspirin to prevent embolic events in IE patients and improve outcome. However, the results of these studies are controversial (Chan et al., 2003, 2008; Anavekar et al., 2007; Eisen et al., 2009; Pepin et al., 2009; Snygg-Martin et al., 2011; Habib et al., 2013) and the clinical usefulness of antiplatelet approaches in IE has been questioned.

We will describe hereafter recent advances on the potential benefits of the platelet P2Y12 receptor inhibitor ticagrelor in IE in regard to data that have previously been obtained with aspirin.

## Why Could Targeting Platelets Be Beneficial Against *Staphylococcus Aureus* Infective Endocarditis?

Besides their primary role in thrombosis and hemostasis, it is now well established that platelets also act as first-line immune responders following pathogen invasion (Holinstat, 2017). Platelets express Toll-like receptors (TLRs), enabling them to recognize pathogen-associated molecular patterns (PAMPs). They can target and fight pathogens via the release of antimicrobial peptides from platelet α-granules, including defensins, thrombocidins, and kinocidins (Yeaman, 2010; Wong et al., 2013). Furthermore, they can communicate with, and modulate the function of other immune cells through the release of immunomodulating mediators (Wong et al., 2013; Gaertner et al., 2017; Surewaard et al., 2018). Platelets have been reported to be essential in transporting bacteria to the hepatic Kupffer cells via a “touch-and-go” mechanism, mediating macrophage-induced clearance of bacteria from the blood stream (Wong et al., 2013).

**FIGURE 1:**
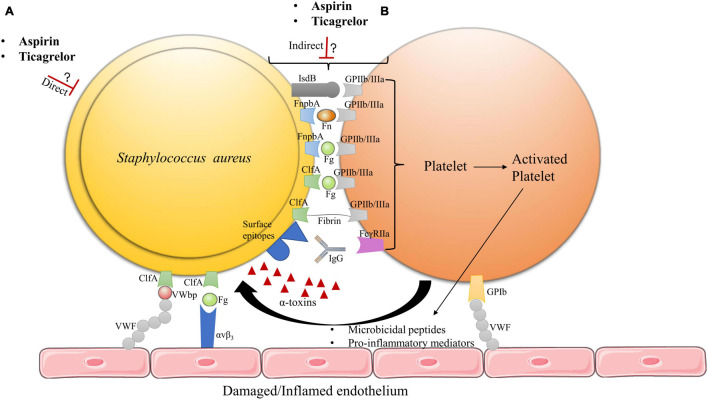
Hypothetical model of aspirin and ticagrelor action on *S. aureus* IE vegetation. **(A)** The antiplatelet drugs may exert direct effects on bacteria. **(B)** Ticagrelor and/or aspirin may inhibit bacteria–platelet interactions by acting on either platelet activation or bacteria toxins, which could also alter the adhesion of these hetero-aggregates on damaged or inflamed endothelia. The exact mechanisms of action of aspirin and ticagrelor on vegetation formation remain to be determined, as well as potential added value of ticagrelor compared to aspirin. FnbpA, fibronectin binding protein A; ClfA, clumping factor A; IsdB, iron regulated surface determinant B; Fn, fibronectin; Fg, fibrinogen; IgG, immunoglobulins; VWF, von Willebrand factor; VWbp, von Willebrand factor binding protein (Claes et al., 2017, 2018; Liesenborghs et al., 2018).

Because inhibiting platelets may prevent them to exert their antimicrobial activity, we need to better understand why and how targeting platelets could be beneficial in bacterial infectious diseases such as IE. The initial phase of IE development involves the interplay between platelets and bacteria (Figure 1; Hamzeh-Cognasse et al., 2015; Liesenborghs et al., 2018, 2019), which strongly suggests an essential role for platelets in early stages of IE. Vegetations could form either via indirect or direct interaction of bacteria with platelets (Hamzeh-Cognasse et al., 2015; Hannachi et al., 2020; Figure 1).

*S. aureus –*bacteria interactions are mainly mediated through binding of the GPIIb/IIIa platelet receptor (integrin a_IIb_b_3_) (Hamzeh-Cognasse et al., 2015; Liesenborghs et al., 2018, 2020). Direct interaction of *S. aureus* with platelet GPIIb/IIIa can occur via the bacterial iron regulated surface determinant B (IsdB) receptor (Liesenborghs et al., 2018). Indirect interaction of *S. aureus* to platelets occurs via several surface membrane motifs referred to as microbial surface components recognizing adhesive matrix molecules (MSCRAMM’s), which comprise fibronectin binding protein A (FnbpA) and clumping factor A (ClfA) (Kerrigan et al., 2008; Cox et al., 2011; Liesenborghs et al., 2018). During IE development, ClfA would be essential for early valve colonization whereas FnbpA would be required for persistent infection and further disease progression (Kerrigan et al., 2008; Liesenborghs et al., 2018). MSCRAMM’s can bind plasma proteins, which enables bridging of *S. aureus* to platelets, mainly via GPIIb/IIIa (Pawar et al., 2004; Kerrigan et al., 2008; Liesenborghs et al., 2018). While ClfA binds with a high affinity to soluble and immobilized fibrinogen (Fg) as well as fibrin, FnbpA binds to both Fg and Fibronectin (Fn), but, with a higher affinity for Fn (Wann et al., 2000; Claes et al., 2018; Liesenborghs et al., 2018). In addition to GPIIb/IIIa platelet receptor activation, bridging of *S. aureus* via immunoglobulins (IgG) to the FcγRIIa platelet receptor is required to induce full aggregation of platelets (Liesenborghs et al., 2018). MSCRAMM’s are also essential in mediating bacterial adhesion to the heart valve surface. Such adhesion is achieved via direct binding of *S. aureus* to endothelial exposed von Willebrand factor (VWF) through the bacteria-secreted von Willebrand factor binding protein (VWbp) and bridging of *S. aureus* to the αvβ_3_ endothelial integrin via Fg. Furthermore, bacteria can use platelets to adhere to the endothelium despite the high shear stress that is encountered at heart valves (Claes et al., 2017, 2018; Liesenborghs et al., 2018, 2019). Based on these mechanisms, it is possible that inhibiting the aspirin-sensitive or the P2Y12 receptor ADP-dependent amplification pathways of platelet activation, downstream of GPIIb/IIIa, could impact platelet–bacteria interactions and eventually, IE development.

Bacteria like *S. aureus* are capable of developing several virulence mechanisms to counteract platelet antimicrobial activity (Hamzeh-Cognasse et al., 2015; Liesenborghs et al., 2020). These mechanisms may allow bacterial survival in the bloodstream and contribute to the development of endovascular infectious diseases such as IE. Of interest is the secreted *S. aureus* α-toxin (Cox et al., 2011; Hamzeh-Cognasse et al., 2015). This pore-forming protein, encoded by the *Hla* gene, interacts with several eukaryotic cell types, including myeloid cells, platelets, and endothelial cells via its disintegrin and metalloproteinase 10 (ADAM10) receptor (Powers et al., 2015; Surewaard et al., 2018). At sub-cytolytic concentration, α-toxin binding to ADAM10 induces proteolysis of VE-cadherin, causing activation of the endothelium (Powers et al., 2012). On platelets, the sub-cytolytic concentration of α-toxin induces ADAM10-mediated cleavage of the platelet GPVI receptor, which hampers platelet adhesion and aggregation. In contrast, at cytolytic concentration, α-toxin causes aberrant platelet activation and aggregation (Bhakdi et al., 1988; Surewaard et al., 2018). Accordingly, observations by Bhakdi et al. (1988) and Bayer et al. (1997) indicate that α-toxin can promote the formation of IE thrombi. α-toxin also enables bacteria to evade platelet antimicrobial activity and cause activation of pro-inflammatory pathways (Powers et al., 2015; Surewaard et al., 2018). Recently, Sun et al. (2021) reported α-toxin to induce the release of endogenous platelet sialidase, resulting in desialysation of platelet glycoproteins and β-galactose exposure. This process accelerates platelet clearance by the hepatic Ashwell-Morell receptor (AMR), which is responsible for *S. aureus* bacteremia-associated thrombocytopenia (Sørensen et al., 2009; Surewaard et al., 2018; Sun et al., 2021).

Thus, preventing α-toxin from inhibiting platelet antimicrobial activity could also be considered as part of the antiplatelet approach against IE.

## Aspirin or Ticagrelor?

*In vitro* studies focusing on the effect of aspirin on platelet–*S. aureus* interactions found that its main metabolite salicylic acid (SAL) regulates the expression of *S. aureus* genes encoding for virulence factors (Kupferwasser et al., 1999a). SAL has been linked to an overexpression of the sigma factor B operon, resulting in the repression of staphylococcal accessory regulator A (Sar A) and accessory gene regulator (Agr). By repressing Sar A and Agr, SAL can diminish the expression of MSCRAMM’s and α-toxin secretion (Kupferwasser et al., 2003; Gordon et al., 2013). The reduced expression of virulence factors could result in slowing down vegetation growth by decreasing platelet–bacteria interactions, thereby enhancing the antimicrobial activity of platelets. More particularly, the inhibition of α-toxin secretion could delay α-toxin enhanced platelet clearance via the hepatic AMR pathway, preserving platelet function (Surewaard et al., 2018; Sun et al., 2021).

Several studies have been performed in different animal models of IE in order to analyze the effect of aspirin on vegetation growth (Table 1). Kupferwasser et al. (1999b) described a significant reduction in bacterial density and vegetation weight using a prophylactic therapy of 8 mg/kg aspirin in a rabbit model of *S. aureus* IE (SAIE). Furthermore, this study indicated that pre-treatment of *S. aureus* with SAL reduced the ability of bacteria to adhere to vegetations (fibrin-platelet surface) (Kupferwasser et al., 1999b). Another study in rabbits showed a key role for both Sar A and the stress response gene *sigB* in mediating the antistaphylococcal effects of SAL *in vivo* (Kupferwasser et al., 2003). In contrast, studies by Nicolau et al. (1999) and Veloso et al. (2015b) described no reduction of vegetation weight when preventively using 10 and 8 mg/kg of aspirin as a monotherapy in a rabbit and rat model of IE, respectively. While Nicolau et al. (1999) reported this effect to be related to the low sample size of the study, Veloso et al. (2015b) stated a possible effect of bolus injection of bacteria in previous models, which induced transient bacteremia, thus negating the effect of preventive antiplatelet therapy. However, Veloso et al. (2015b) could observe a significant decrease in vegetation weight when using aspirin in combination with ticlopidine, another antiplatelet drug belonging to the thienopyridine class of platelet P2Y12 receptor inhibitors. Finally, a combination of aspirin with vancomycin was described to significantly decrease vegetation weight and bacterial density, emphasizing its potential as an adjuvant therapeutic agent (Nicolau et al., 1995; Veloso et al., 2015b).

**TABLE 1:** Overview of pre-clinical and clinical studies on the use of antiplatelet therapy in the prevention of infective endocarditis.

**Author, year**	**Type of study**	**Study model**	**Outcomes**
**Pre-clinical studies**			
Nicolau et al., 1995	*In vivo*	SAIE Rabbit	-Reduced vegetation weight and bacterial density using a prophylactic dose of 10 mg/kg aspirin -Reduced vegetation weight and rate of sterilization using early adjuvant treatment with 10 mg/kg aspirin + 50 mg/kg vancomycin
Kupferwasser et al., 1999b	*In vivo*	SAIE Rabbit	-Reduced vegetation weight, vegetation/renal bacterial densities, and renal embolic lesions using a prophylactic dose of 8 mg/kg aspirin -Reduced *S. aureus* adhesion to vegetation (platelet-fibrin matrix) when pre-exposed to SAL
Nicolau et al., 1999	*In vivo*	SAIE Rabbit	-Reduced vegetation weight and infected vegetations, using a prophylactic dose of 10 mg/kg aspirin + 10 mg/kg ticlopidine -No reduced vegetation weight using a prophylactic dose of 10 mg/kg aspirin
Veloso et al., 2015b	*In vivo*	SAIE Rat	-Reduced vegetation weight and infected vegetations using a prophylactic dose of 8 mg/kg aspirin + 10 mg/kg ticlopidine -No reduced vegetation weight using a prophylactic dose of 8 mg/kg aspirin
**Clinical studies**			
Chan et al., 2003	Prospective, randomized, double-blinded, placebo-controlled trial	IE patients receiving prior aspirin therapy	-No reduced rate of embolic events -Increased bleeding
Anavekar et al., 2007	Retrospective cohort trial	IE patients receiving prior antiplatelet therapy	-Reduced rate of embolic events
Chan et al., 2008	Prospective, multi-center, randomized trial	IE patients receiving prior aspirin therapy	-No reduced rate of embolic events -Increased bleeding risk
Eisen et al., 2009	Prospective cohort trial	SAIE patients receiving prior aspirin therapy	-Reduced valve replacement surgery -No reduced rate of embolic events
Pepin et al., 2009	Retrospective observational trial	IE patients receiving prior antiplatelet therapy	-No reduced rate of embolic events
Snygg-Martin et al., 2011	Prospective cohort trial	IE patients receiving prior antiplatelet therapy	-No reduced rate of cerebrovascular complications
Habib et al., 2013	Retrospective trial	Cardiovascular implantable electronic device IE patients receiving prior aspirin therapy	-Reduced rate of embolic events

*SAIE, *S. aureus* infective endocarditis; IE, infective endocarditis.*

Clinical studies focusing on prior aspirin therapy in patients at high risk of IE described variable outcomes in relation to the prevention of embolic events (Table 1). Chan et al. (2003, 2008) reported no benefit of aspirin in reducing the risk of embolic events in IE patients, however increased bleeding was observed. This was further confirmed by Snygg-Martin et al. (2011), Eisen et al. (2009), and Pepin et al. (2009), showing no reduction of cerebrovascular complications or embolic events in patients on previously established antiplatelet therapy (mostly aspirin) (Trauer et al., 2017). In contrast, Anavekar et al. (2007) and Habib et al. (2013) described prior aspirin therapy, to reduce vegetation formation and embolic events. Despite promising mechanistic *in vitro* and animal studies, clinical studies showed controversial results. Thus, there is currently no evidence for any benefits of antiplatelet drugs such as aspirin in improving IE patient outcome. Nevertheless, many of these clinical studies had a low sample size which made it difficult to obtain sufficient statistical power. Furthermore, there is a large heterogeneity in patient age, comorbidities, the duration and dose of antiplatelet therapy prior to IE development or after, and bacterial strains involved in disease development.

In contrast, the relatively more recent antiplatelet drug ticagrelor, a reversible platelet P2Y12 receptor inhibitor, has become subject of discussion. In a sub-study of the large, randomized PLATO clinical trial, ticagrelor therapy was associated with a lower risk of death related to infection as compared to the thienopyridine clopidogrel. In addition, the small XANTHIPPE clinical study showed improved lung function in pneumonia patients treated with ticagrelor (Wallentin et al., 2009; Sexton et al., 2018; Lupu et al., 2020). The study by Lancellotti et al. (2019a) demonstrated bactericidal activity of ticagrelor and its main metabolite against Gram-positive bacteria such as methicillin-susceptible *S. aureus* (MSSA) and *E. faecalis*, as well as Gram-positive resistant strains, including methicillin-resistant *S. epidermidis* (MRSE), methicillin-resistant *S. aureus* (MRSA), and Vancomycin-resistant Enterococcus (VRE). Importantly, these effects were not observed with the active metabolite of prasugrel, another thienopyridine P2Y12 inhibitor (Wallentin et al., 2009; Lancellotti et al., 2019a).

The reported *in vitro* antibacterial concentration (Minimum inhibitory concentration value) of ticagrelor against MSSA and MRSA was around ten times higher than the recommended antiplatelet dosage (Lancellotti et al., 2019a). However, the use of a mouse model implanted with an *S. aureus*-infected subcutaneous disc supported the antibacterial effect of ticagrelor at antiplatelet dosage, as shown by a significant decrease of *S. aureus* biofilm growth on implants and dissemination of bacteria to surrounding tissues (Lancellotti et al., 2019a). Although systemically, bactericidal concentrations are not reached *in vivo*, bactericidal activity at the infection site could still be achieved at antiplatelet dosage through an unknown mechanism, hypothesized to be platelet related (Lancellotti et al., 2019a). Recently, a preclinical and *in vitro* study has been performed focusing on the role of ticagrelor in eradicating *S. aureus* bacteremia and preserving the ability of platelets to kill bacteria (Sun et al., 2021; Ulloa et al., 2021). Ulloa et al. (2021) described successful use of ticagrelor as an adjuvant therapy to antibiotics in a case report of a male patient with MSSA bacteremia and thrombocytopenia. The patient received antibiotic treatment but remained bacteremic. On day five, ticagrelor was administered which resulted in a decreased, non-detectable bacterial blood count and an increase in platelet count to a low-normal range (Ulloa et al., 2021). Furthermore, the case report result was supported by an *in vitro* study, showing that ticagrelor could prevent α-toxin-induced inhibition of platelet antibacterial activity (Sun et al., 2021; Ulloa et al., 2021). Indeed, *in vitro*, platelet pre-treatment with ticagrelor improved *S. aureus* killing (Sun et al., 2021; Ulloa et al., 2021). However, the mechanism of such an effect remains unclear.

To date, no studies have investigated the potential effect of prior ticagrelor therapy in preventing IE development.

## Discussion

The use of antiplatelet drugs as an adjunct therapy to prevent vegetation growth, embolic events, or to improve the outcome in high-risk cardiovascular patients with IE has been and should still be a matter of great interest. While the mode of action and possible benefits of aspirin in the prevention of IE progression have been widely investigated, the more recent antiplatelet drug ticagrelor deserves attention. Several hypotheses have been proposed regarding its antibacterial properties. Lancellotti et al. (2019b) reported a possible role of platelets for ticagrelor transport to the site of infection, allowing a local antibacterial effect. This hypothesis is based on the reversible binding properties of ticagrelor to the P2Y12 receptor, and on studies indicating that platelets are recruited to the site of infection, similar to immune cells (Lancellotti et al., 2019b). Heying et al. (2019) proposed that the bactericidal properties of ticagrelor could resemble the aspirin effect, modulating the expression of *S. aureus* virulence factors with a decrease in the expression of MSCRAMM’s and toxins. The potential inhibitory effect of ticagrelor on bacteria-platelet interactions was further supported by an *in vitro* study reporting the highest inhibitory effect of bacteria-induced platelet aggregation by ticagrelor as compared to aspirin, aspirin plus ticagrelor, or tirofiban (Hannachi et al., 2020). Very recently, two studies described an inhibitory effect of ticagrelor on α-toxin mediated platelet clearance by the hepatic AMR pathways, thereby preserving the antibacterial activity of platelets (Sun et al., 2021; Ulloa et al., 2021). In addition, antiplatelet drugs could also inhibit the immune and inflammatory role of platelets (Tiwari et al., 2020). While aspirin inhibits the release of inflammatory mediators by platelets such as leukotrienes, ticagrelor blocks the formation of platelet-leukocyte aggregates (Tiwari et al., 2020), which could also play a role during the process of infection, as proposed by Sexton et al. (2018).

## Conclusion

Antibiotic prophylaxis is currently recommended to prevent IE development in high-risk patients. However, the use of antibiotics faces the challenge of a low efficacy due to the steadily increasing infection rate by resistant bacteria strains, which is further enhanced by using antibiotics. This review suggests that the antiplatelet drug ticagrelor combined with antibiotics may play a role in the prevention of SAIE. Indeed, this drug was recently described to have antibacterial properties in addition to its potent antiplatelet activity. Moreover, a recent study in mice and a case report remarkably indicated the ability of ticagrelor to eradicate *S. aureus* bacteremia. Therefore, further investigations should be performed in order to evaluate whether prior ticagrelor therapy could be beneficial for the prevention of IE or other endovascular infectious diseases. This new strategy could contribute to a decrease in antibiotic resistance and a significant reduction in disease-associated mortality.

## Author Contributions

All authors listed have made a substantial, direct and intellectual contribution to the work, and approved it for publication.

## Conflict of Interest

The authors declare that the research was conducted in the absence of any commercial or financial relationships that could be construed as a potential conflict of interest.

## Publisher’s Note

All claims expressed in this article are solely those of the authors and do not necessarily represent those of their affiliated organizations, or those of the publisher, the editors and the reviewers. Any product that may be evaluated in this article, or claim that may be made by its manufacturer, is not guaranteed or endorsed by the publisher.
